# Nonmonotonous temperature dependence of Shapiro steps in YBCO grain boundary junctions

**DOI:** 10.3762/bjnano.12.95

**Published:** 2021-11-23

**Authors:** Leonid S Revin, Dmitriy V Masterov, Alexey E Parafin, Sergey A Pavlov, Andrey L Pankratov

**Affiliations:** 1 Institute for Physics of Microstructures of RAS, GSP-105, Nizhny Novgorod, 603950, Russia; 2 Center of Quantum Technologies, Nizhny Novgorod State Technical University, Nizhny Novgorod, Russia; 3 Lobachevsky State University of Nizhny Novgorod, Nizhny Novgorod, Russia

**Keywords:** characteristic frequency, Shapiro steps, temperature dependence, YBaCuO Josephson junction

## Abstract

The amplitudes of the first Shapiro steps for an external signal with frequencies of 72 and 265 GHz are measured as function of the temperature from 20 to 80 K for a 6 μm Josephson grain boundary junction fabricated by YBaCuO film deposition on an yttria-stabilized zirconia bicrystal substrate. Non-monotonic dependences of step heights for different external signal frequencies were found in the limit of a weak driving signal, with the maxima occurring at different points as function of the temperature. The step heights are in agreement with the calculations based on the resistively–capacitively shunted junction model and Bessel theory. The emergence of the receiving optima is explained by the mutual influence of the varying critical current and the characteristic frequency.

## Introduction

High-temperature superconducting (HTSC) Josephson junctions (JJs) are of great interest since many physical properties can be observed in dynamics during the changing the temperature within a wide range from nitrogen temperatures down to sub-kelvin, such as the phase diffusion regime [1–3], evidence for a minigap [4], and low-noise nano-junctions [5]. Such abilities raise not only fundamental interest in HTSC JJs but also an active search for ways to practically use such JJs. In recent years, the limiting characteristics of detectors and mixers based on HTSC JJs [6–11] have been actively studied. Josephson junctions have also been used for various spectroscopic applications [12]. In this area, the AC Josephson effect is utilized for the Hilbert-transform spectral analysis [13–14].

It should be noted that the simplest marker of the response level of a Josephson junction to microwave (MW) radiation is the magnitude of Shapiro steps. In the majority of works, an increase in sensitivity at low temperatures has been demonstrated [15–17], although a part of the papers indicate the receiver’s operation optimum at intermediate temperatures between the liquid nitrogen and helium temperatures [18–19]. The issue of obtaining sharp Shapiro steps is especially important for the development of HTSC Josephson voltage standards, consisting of series arrays of up to tens of thousands Josephson junctions [20–21]. Biased at frequencies in the range of ω/(2π) = 70–90 GHz, such arrays provide accurate quantized voltages *V_n_* = *n*ℏω/(2*e*) exceeding 10 V. This accuracy is particularly determined by the magnitude of the response to external radiation. The Shapiro step observation can also be used as a clear probe to the gap symmetry of multigap superconductors [22].

The heights of the MW-induced voltage steps have been measured as a function of the MW power for various Josephson weak links fabricated from high-*T*_c_ superconductors [16,23–24]. The measured amplitudes are often smaller than those predicted by the resistively–capacitively shunted-junction (RCSJ) model [25–26], especially for measurements obtained at high temperatures. However, taking into account the effect of the YBCO junction resistance thermal noise [16] makes it possible to neutralize this difference and obtain a good agreement between the experiment and the theory.

While for low-temperature JJs the temperature dependence of the Shapiro steps is weak [27], for HTSC junctions the response to a MW signal has a general tendency to rise with decreasing temperature, but may have peculiarities for certain sample parameters [19].

In this paper, we investigate the temperature dependence of the first Shapiro step amplitude for an external signal with frequencies of 72 and 265 GHz acting on YBa_2_Cu_3_O_7−δ_ 6 μm Josephson grain boundary junction. The observed non-monotonous behavior of the step height in the limit of low signal power is discussed, and the measurement results are compared with the results of numerical calculations.

## Experimental Setup and Numerical Model

The samples of grain boundary Josephson junctions were fabricated by on-axis dc magnetron sputtering [28–31] of YBa_2_Cu_3_O_7−δ_ (YBCO) film on the surface of 24°[001]-tilt Zr_1_*_−x_*Y*_x_*O_2_ bicrystal substrates with modification of the substrate surface by preliminary topology masks [29–30]. The junctions with length *L* = 6 μm along the grain boundary and thickness 0.3 μm were integrated into a dipole antenna. The structure look follows the design from [29]. Based on the analysis of the transport properties, the best structure was selected and located at the center of a Si lens for efficient detection. The sample was mounted into a dry cryostat allowing for measurements in a wide temperature range from helium temperatures to ≈80 K. An external gigahertz signal was fed through an optical window with IR filters using a semiconductor synthesizer with a multiplier (70–78 GHz) or using a backward wave oscillator (230–370 GHz). The JJ transport properties and the response were characterized by a precise Keithley low-noise current source and nanovoltmeter using a standard 4-probe technique.

In the RCSJ model to which we compare our experimental results, the junction phase ϕ with an ideal critical current *I*_c_, a resistance *R*_N_ and a capacitance *C* are described by the stochastic differential equation [32–33]


[1] $$I=I_{\text{c}}\sin\phi +\frac{V}{R_{\text{N}}}+C\frac{\text{d}V}{\text{d}t}+I_{\text{mw}}\sin\left(2\pi F_{\text{mw}}t\right)+I_{\text{F}},$$

where the voltage *V* = dϕ/d*t* × 2π/Φ_0_ (Φ_0_ is the magnetic flux quantum). The thermal fluctuations *I*_F_ are assumed to be a white Gaussian noise with zero mean and correlation function




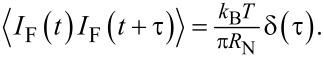




A simple harmonic signal of the amplitude *I*_mw_ and the frequency ω_mw_ = 2π*F*_mw_ describes an external high-frequency radiation of the power 
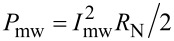
. Its effect on the Josephson system particularly depends on the characteristic frequency ω_c_ = 2*eI*_c_*R*_N_/ℏ of the JJ.

## Results

**Figure 1:**
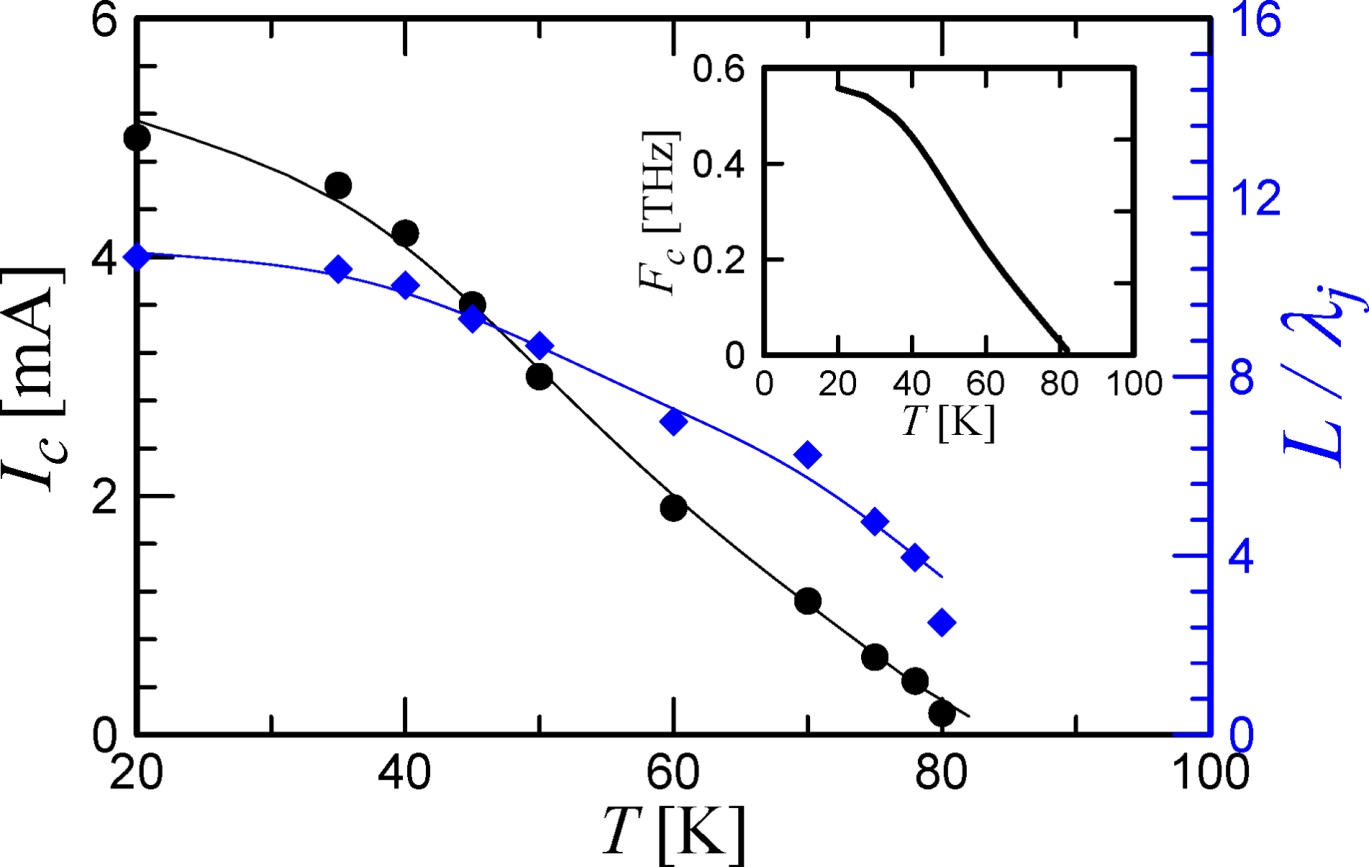
The dependence of the critical current (black dots) and the characteristic length of the Josephson junction (blue diamonds) on the temperature. The solid curves are spline approximations. The inset shows *F*_c_ = ω_c_/(2π) versus *T*.

First, the current–voltage characteristics (IVCs) were measured, and the value of the critical current as a function of temperature was found, see Figure 1. The *I*_c_(*T*) dependence is similar to the experimental observations for other such structures [34–36]. At the same time, the normal resistance of the JJ remained virtually constant, that is, *R*_N_ was 0.23–0.24 Ω within the whole studied temperature range. For the subsequent analysis of the results, we used data from the literature about similar structures of an YBCO bicrystal junction on 24°[001]-tilt Zr_1_*_−x_*Y*_x_*O_2_ substrate [37] as the value of the junction capacitance *C* = 3 × 10 ^−2^ F/m^2^ × 1.8 × 10^−12^ m^2^ = 0.05 pF. This value, according to [35], remains almost unchanged over a wide temperature range.

It is important to understand which parameters vary in the model with the temperature. Figure 1 also shows the change in the Josephson junction characteristic length *L*/λ_J_, where 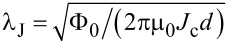 is the Josephson penetration depth, which determines the size of a fluxon in the junction. Here μ_0_ is the vacuum permeability, *J*_c_ is the critical current density, and *d* = *t* + 2λ_L_ is the effective magnetic thickness with the junction barrier thickness *t* = 1.5 nm and the London penetration depth λ_L_ = 250–150 nm [38]. It can be seen from the figure that, for nitrogen temperatures, the Josephson junction can generally be considered as a short JJ. With the decrease in the temperature, its characteristic dimension increases, and for 20 K, in the general case, Equation 1 becomes invalid, that is, the dynamics of the spatial distribution of the phase and the magnetic field inside the junction becomes important [39–41]. In the case of long JJs it is necessary to consider the sine-Gordon equation, taking into account the non-uniform distribution of currents flowing through the barrier, which is typical for bicrystal junctions [28,42–43]. However, if the junction length is of the order of the kink size and there is no external magnetic field, the long junction dynamics is close to that of a short one [39] and the used model is qualitatively adequate. This is confirmed in [40], where the escape time from the superconducting state is investigated, and it is shown that the critical length *L*/λ_J_ = 5 corresponds to the crossover between two dynamical regimes. Nevertheless, long HTSC junctions are characterized by such features as a flux creep and the change in the IVC curvature associated with the crossover from the flux flow to Josephson junction behavior [44]. That is why, as it will be shown below, in the region of low temperatures, the agreement between the experiment and the numerical calculation is not as good as in the region of high *T* values.

The second important parameter is the characteristic frequency ω_c_ (or *F*_c_) (see the inset of Figure 1). The change of ω_c_ radically affects the response of the system to an external MW signal [17]. Essentially, the ω_mw_/ω_c_ (or *F*_mw_/*F*_c_) ratio determines if the detection regime is optimal for the junction. This issue is discussed in more details below.

The third important parameter is the thermal noise magnitude, *k*_B_*T*, which affects the smearing of the Shapiro steps, and, accordingly, the decrease in the step size in the region of low radiation power. It is not shown in Figure 1.

**Figure 2:**
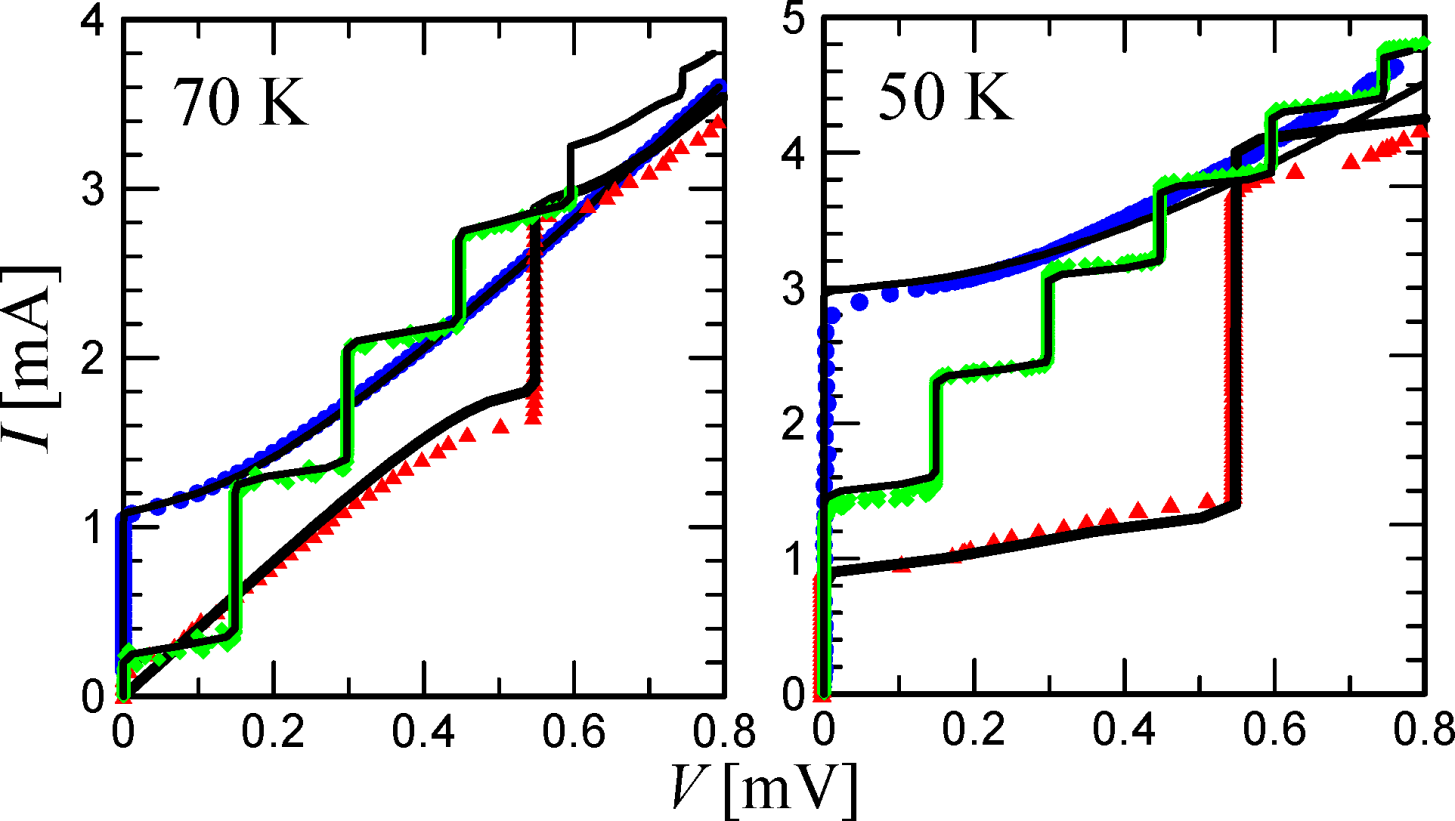
IVCs of a Josephson junction without an MW signal (blue dots), under the action of an external signal of 72 GHz (green diamonds) and 265 GHz (red triangles) at temperatures of 70 and 50 K. The black lines are the numerical calculations for each curve with the experimental parameters and with fitting power *P*_mw_.

Figure 2 shows the IVCs for temperatures of 70 and 50 K in the absence of a high-frequency signal and in the regime of detecting external 72 or 265 GHz signals. The measurement results are in good agreement with the numerical simulations (the black curves). It should be noted that the radiation power was the same for the measurements at all temperatures. The power level of the two signals, 72 and 265 GHz, was chosen to be near the first minimum of the critical current, and, accordingly, near the first maximum of the first Shapiro step at high temperatures. This can be seen from the IVC for *T* = 70 K and *F*_mw_ = 72 GHz: the critical current is nearly zero, the amplitude of the first step is greater than the amplitude of the second and the third steps. The same picture is observed for the IVC at *F*_mw_ = 265 GHz. The comparison with the numerical model gives an estimate of the power absorbed by the Josephson junction: it is 0.4 μW for 72 GHz, and *P*_mw_ = 3 μW for 265 GHz.

**Figure 3:**
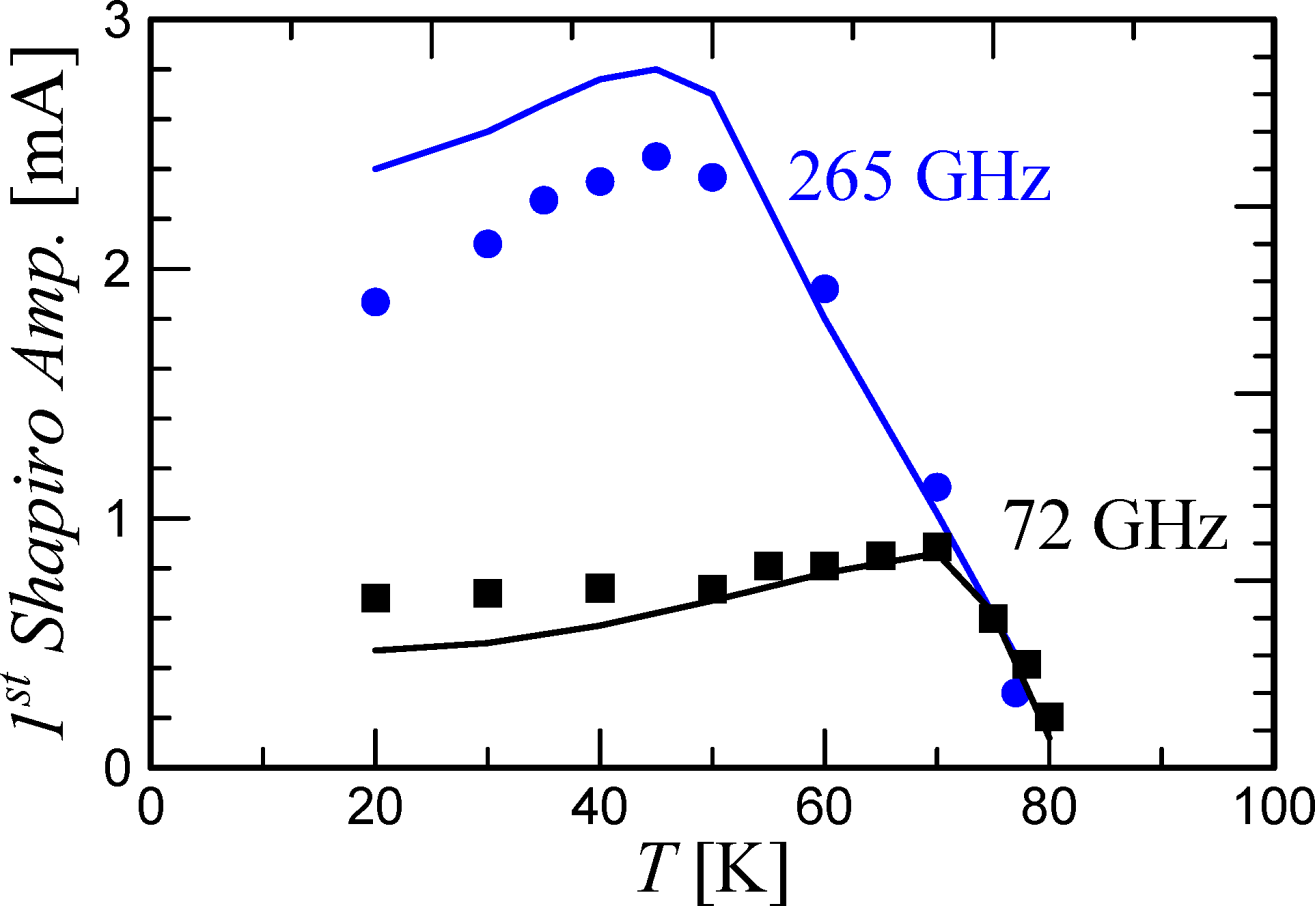
The dependence of the first Shapiro step amplitude on the temperature for 72 and 265 GHz radiation at a constant power. The dots are the experimental values, the lines are the theory for the temperatures at which the measurements were conducted.

Figure 3, essentially the main result of the article, demonstrates the dependence of the first Shapiro step amplitude on the temperature for 72 and 265 GHz radiation at a constant power. The dependences are non-monotonic and have a maximum located at different temperature values for different MW frequencies. In addition, it can be seen that at high temperatures of approx. 80 K, the amplitudes of the steps are close, while with decreasing temperature in the case of 265 GHz radiation, the Shapiro steps become significantly higher than for 72 GHz. The numerical results (the solid curves) based on the experimental data describe the experiment at high temperatures well and differ quantitatively at low temperatures. This may be caused by a specific dynamics arising with an increase in the characteristic length of the JJ at low temperatures. Nevertheless, the simulation qualitatively follows the experimental dependence within the entire temperature range.

The obtained effect of the optimum in the JJ response is associated with a simultaneous change of several parameters when the temperature changes. For a qualitative analysis, let us consider the expression for the first Shapiro step amplitude [33,45–46]:


[2] $$\Delta I_{1}=I_{\text{c}}\sum_{k=-\infty }^{+\infty }J_{k}\left(a\right)J_{-1-k}\left(a\right)\left|I_{\text{p}}\left[\left(k-\frac{1}{2}\right)\omega_{\text{mw}}\right]\right|,$$

where *J_k_* and *J*_−1_*_−k_* are Bessel functions at 
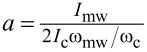
, *I*_p_ is a complex function that determines the quadrature components of the supercurrent depending on the Josephson generation frequency. Although this expression is valid for a voltage-biased JJ, it is in a good agreement with measurements for the current-biased regime and RCSJ model [35]. In the case of low signal power and ω_mw_ ≪ ω_c_, the maximum height of the first step is proportional to


[3] $$\max\Delta I_{1}\approx I_{\text{c}}\omega_{\text{mw}}/\omega_{\text{c}}.$$

In the limit of ω_mw_ ≈ ω_c_, the expression for Δ*I*_1_ takes the simple form:


[4] $$\Delta I_{1}\approx 2I_{\text{c}}\left|J_{1}\left(\frac{I_{\text{mw}}}{2I_{\text{c}}\omega_{\text{mw}}/\omega_{\text{c}}}\right)\right|.$$

**Figure 4:**
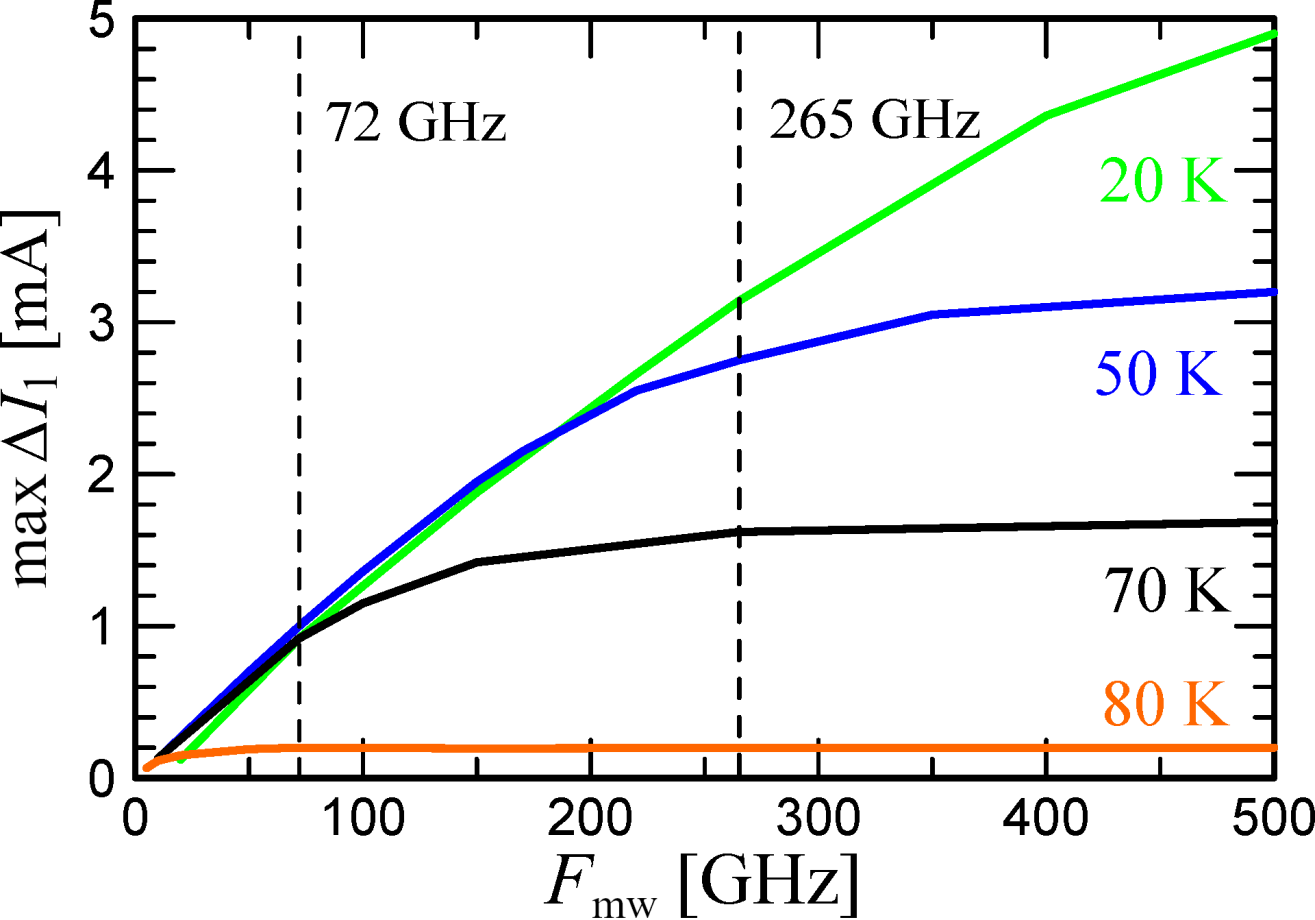
max Δ*I*_1_ as function of *F*_mw_ at various temperatures. The dotted lines mark the position of the two frequencies used in the experiment.

Figure 4 shows the theoretical dependence of maxΔ*I*_1_ on the frequency for various temperatures. According to Equation 3, the maximum step amplitude increases as the critical current increases and the temperature goes down. At the same time, due to the change in the critical frequency ω_c_ (the inset in Figure 1), the optimal signal detection regime is shifted. That is, for temperatures of 80 K and 70 K and the frequency of 72 GHz, the condition ω_mw_ ≈ ω_c_ is satisfied, and the step heights reach ≈*I*_c_ and ≈0.9 *I*_c_, respectively. At 50 K, maxΔ*I*_1_ ≈ *I*_c_ω_mw_/ω_c_ = *I*_c_*F*_mw_/*F*_c_ = 3 mA × 72 GHz/330 GHz = 0.65 mA, and at 20 K, maxΔ*I*_1_ ≈ 5 mA × 72 GHz/560 GHz = 0.64 mA. For 265 GHz signal, the step height almost reaches the limit ≈*I*_c_ at 50 K, while at 20 K, ω_mw_ is still far from ω_c_. Summarizing, for low-gigahertz radiation frequencies, lowering the temperature does not gain the response magnitude due to the non-optimal frequency of signal detection. Whereas, the closer ω_mw_ to the characteristic frequency, the greater the influence of the critical current increase with the temperature takes place.

**Figure 5:**
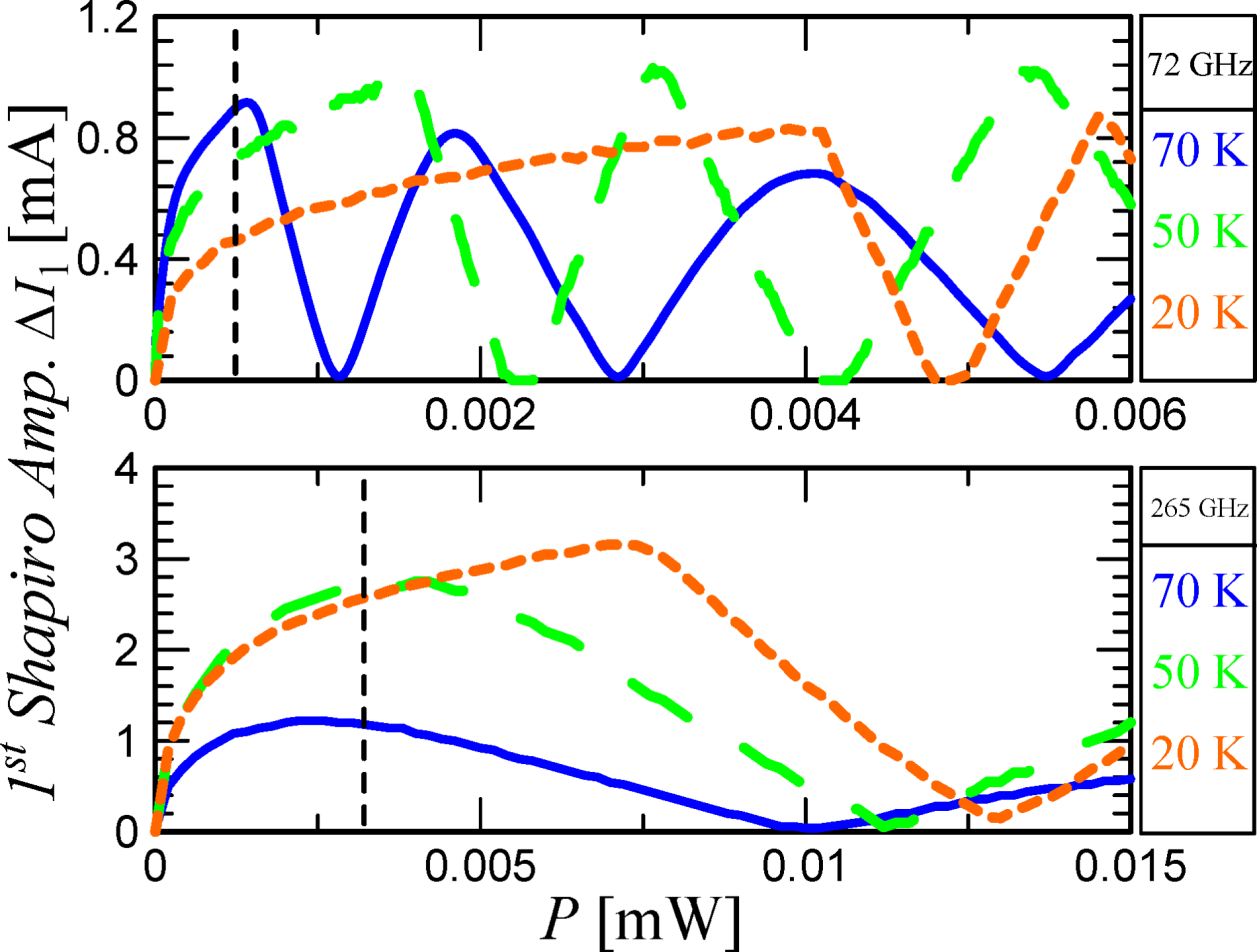
The first Shapiro step as function of *P*_mw_ at three temperatures and under a signal at 72 GHz (upper graph) and 265 GHz (lower graph). The black dashed lines indicate the power levels from the experiment.

In addition to the magnitude of the Shapiro step height maximum, it is important to take into account the period of the Bessel function, which, in the first approximation, determines the response of the JJ to a change in the gigahertz-signal power. Equation 4 shows that as ω_c_ grows, the Bessel function period increases, that is, the derivative dΔ*I*_1_/d*P*_mw_ decreases. Figure 5 shows the results of the numerical calculations of the first Shapiro step height versus the external signal power at the temperatures of 70, 50, and 20 K. The upper panel of Figure 5 corresponds to the external signal frequency of 72 GHz. It can be seen that maxΔ*I*_1_ is close for all three temperatures, as explained earlier, see Figure 4. Nonetheless, due to the shift in the step maximum position in power, for small signal levels (marked with a vertical dashed line), Δ*I*_1_ at 70 K is larger than at 50 and 20 K. The bottom panel of Figure 5 corresponds to a 265 GHz external signal. Here, for different temperatures, there is also a shift in the position of the Shapiro step maximum along the power axis, but it is smaller in comparison with the previous case, since ω_mw_/ω_c_ is closer to unity. In this case, the increase in the maximum step height with temperature is also significant. Nevertheless, there is an optimum Δ*I*_1_ in temperature due to the competition between two effects, namely an increase in maxΔ*I*_1_ with an increase in the critical current and a decrease in dΔ*I*_1_/d*P*_mw_ with an increase in the critical current.

## Conclusion

The response in the form of the amplitudes of the Shapiro steps to an external signal with frequencies of 72 and 265 GHz was measured for 6 μm YBaCuO bicrystal junctions as a function of temperature in the range from 20 to 80 K. Nonmonotonic dependences of the step height were found in the region of a weak external signal with maxima at various points. The heights of the steps are consistent with calculations based on the RCSJ model and are qualitatively described by Bessel functions. The occurrence of the receiving optima is explained by the mutual influence of the varying critical current and the characteristic frequency. The maximum response to a 72 GHz signal has an optimum at 70 K, while to a 265 GHz signal – at 50 K.

For applied tasks of terahertz imaging [47], mixing [36], and Hilbert-transform spectral analysis [13] it is not possible to vary the incident power over a wide range. The power level is set there by losses, mismatch, and power absorption by the samples under study. Moreover, in applied problems one has to deal with low power levels and a linear response of detector [48]. Specifically in this area of the device operation, the effect described in the paper can be observed.

The obtained optima arise at certain JJ parameters (*R*_N_, *I*_c_(*T*), *C*, and ω_c_(*T*)). Depending on these parameters, such maxima may appear [18–19] or not appear [17] in the measurements at an intermediate temperature. For specific purposes and operation regions, it is possible to tune JJ parameters to operate in the optimal regime [47,49]. In addition to JJ characteristics, the operating frequency or the frequency range is important. For low ω_mw_, the change in the response of the Josephson junction will be small with the temperature [16] since at these frequencies the detection is not optimal. At the same time, at high temperatures, thermal noise will blur the step more than at low temperatures, and with increasing *I*_c_ the step height will increase. This also applies to high frequencies close or greater than the gap. Non-monotonous peculiarities in the response will occur at intermediate frequencies at, in fact, the most interesting range from a practical point of view. The same optima of the response can be achieved in the operation temperature range at a low power of the external signal with a higher normal resistance and critical current of the sample.

Therefore, lowering the temperature for the HTSC does not necessarily lead to an improvement in the detection properties of the Josephson junctions. An interesting question for further investigation is the search for an analytical expression for the optimal temperature of receiving an external signal of a given power and frequency for given JJ parameters.
